# An Approach to Balancing the Positive and Negative Effects of Elevated Nitrogen Oxides in the Lower Atmosphere on Terrestrial Plants

**DOI:** 10.1100/tsw.2001.87

**Published:** 2001-09-24

**Authors:** Serguei Semenov

**Affiliations:** Institute of Global Climate and Ecology, Moscow, Russia

**Keywords:** nitrogen oxides, ozone, atmosphere, nitrogen deposition, soil acidification, terrestrial plants productivity

## Abstract

Elevated NO_x_ in the lower atmosphere has three major effects on terrestrial plants. On the one hand, it causes an increase in surface ozone concentration. This reduces plant growth rate. On the other hand, elevated NO_x_ causes an increase in the flux of oxidized N compounds from the atmosphere to the land surface. This plays a dual role in the life of terrestrial plants. Additional N in soils stimulates plant growth (N-fertilization effect), whereas soil acidification may negatively affect plants. A simple empirical model for calculating the overall effect of anthropogenic increase in NO_x_ level has been developed. The model is based on experimental “cause-response” data presented in world scientific literature. Calculations showed that at the large scale, among the above-mentioned changes, elevated O_3_ plays a major and negative role in plant life. Its negative effect on plants is partly compensated by N fertilization in unmanaged ecosystems. Such compensation appears to be negligible in agricultural lands. There are vast territories in Euro-Asia — for instance, a territory of Russia — in which acid atmospheric deposition has no significant effect on terrestrial plants.

## INTRODUCTION

Anthropogenic emissions of nitrogen oxides — nitric oxide (NO) and nitrogen dioxide ($NO_2$), so-called $NO_x$ — are caused mainly by burning processes. The major emission sources are as follows: (1) power plants as well as industrial and municipal enterprises using coal, oil products and natural gas; (2) use of oil products as transport fuels; (3) forest fires and use of wood as a fuel; (4) burning of wastes. Thus, anthropogenic increase in $NO_x$ content in the atmosphere is a consequence of economic development. The major part of anthropogenic $NO_x$ is emitted as NO.

Situations in western and central Europe showed that the increase in $NO_x$ content has become a problem of continental scale in the last two decades of the 20th century[1]. Tables 1 and 2 give an idea of the degree of human-induced disturbance of the global cycle of nitrogen. As one can see from the tables, the overall emissions of nitrogen compounds of some European countries are comparable with natural emissions of whole natural zones, whereas density of emissions are even much higher.

About 95% of human-induced emissions of nitrogen to the atmosphere are released as NO. NO is gradually oxidized there to $NO_2$. Both gases participate in numerous chemical and physical transformations. These substances, as well as some of their derivatives (nitrites, nitrates, nitrous acid), are removed from the atmosphere as a result of wet and dry deposition processes. Such removal can take place in gaseous, liquid, and aerosol phases. The rate of $NO_2$ removal substantially exceeds that of NO.

Enrichment of the boundary layer of the atmosphere with NO initiates two major processes having an effect on terrestrial plants. First, elevated $NO_x$ causes an enhancement of ozone ($O_3$) content. Ozone negatively affects plant growth and reduces productivity. Second, the increase in atmospheric deposition of oxidized nitrogen can lead to two different consequences. On the one hand, there is a positive effect, namely, a sort of nitrogen fertilization of plants, because these substances can be used by plants. On the other hand, deposition of oxidized nitrogen compounds leads to acidification of precipitation and soils that in some cases may negatively affect the mineral nutrition of terrestrial plants.

The main goal of this paper is to propose an approach to balancing the above-mentioned positive and negative effects and to make a preliminary assessment of their contributions to the overall effect at the large scale.

**TABLE 1**
**Estimates of the Global Natural Emissions of NO and $N_2O$ from the Land Surface to the Atmosphere**

| | Annual Emission of NO | | Annual Emission of $N_2O$ | |
|---|---|---|---|---|
| Biome | Amount (Tg* N) | Density (mg N $m^{-2}$) | Amount (Tg N) | Density (mg N $m^{-2}$) |
| Tundra | 0.13 | 10.5 | 0.04 | 2.7 |
| Forest tundra | 0.29 | 36.2 | 0.10 | 10.9 |
| Boreal forests | 0.79 | 48.0 | 0.30 | 17.6 |
| Cultivated lands** | 2.00 | 150.2 | 1.10 | 81.4 |
| Globally from these and other emission sources | 9.69 | — | 6.12 | — |

* Teragram (Tg) = $10^{12}$ g.
** An increase in emissions of N from the land surface to the atmosphere caused by use of fertilizers is not accounted.
Modified from Potter et al.[2].

**TABLE 2**
**Estimates of Anthropogenic Emission of $NO_x$ from the Land Surface to the Atmosphere**

| | Annual Emission of $NO_x$ | |
|---|---|---|
| Country/Region | Amount (Tg N) | Density (mg N $m^{-2}$) |
| Austria* | 0.064 | 765 |
| Netherlands* | 0.16 | 4936 |
| USSR** | 4 | 180 |
| Europe* | 6.56 | 566 |

* Modified from Simpson[3].
** Modified from Ryaboshapko et al.[4].

## METHODS

Figure 1 diagrams the chemical/photochemical reactions between $NO_x$, $O_3$, and peroxyradicals ($RO_2$) to explain a major mechanism for enrichment of the surface layer of the atmosphere with ozone. Peroxyradicals are produced in the lower atmosphere through chemical interactions between radicals of hydroxyl (OH) and Volatile Organic Compounds (so-called VOCs). This latter group is comprised of alkanes, alkenes, aromatic hydrocarbons, alcohols, aldehydes, and some other substances. VOCs can be either natural (e.g., terpenes and isoprene emitted by terrestrial plants) or anthropogenic. Interaction of NO with peroxyradicals leads to conversion of NO into $NO_2$. Further on, photolysis of $NO_2$ leads to ozone enrichment of the atmosphere.

Significant nonlinearity of some of the above-mentioned processes determines certain “surprise” features of the whole system of reactions. For instance, under low levels of VOCs an increase in NO emissions may cause a decline in ozone content[5]. However, in reality, at the large scale, the surface layer of the atmosphere likely contains sufficient amounts of VOCs to ensure a positive correlation between the NO-emission rate and ozone content.

The following reactions play a major role in the processes displayed in this scheme:

$$
\begin{aligned}
NO + O_3 &\rightarrow NO_2 + O_2 \\
NO + RO_2 &\rightarrow RO + NO_2 \\
RO &\rightarrow SINK \\
NO_2 + h\nu &\rightarrow NO + O^{\cdot} \\
O^{\cdot} + O_2 + M &\rightarrow O_3 + M
\end{aligned}
$$

In the latter reaction M means nonreactive molecule absorbing energy.

A mathematical model describing these processes was developed[6] and further adopted as a basic ozone model of the Meteorological Synthesizing Centre-East (MSC-E) of the Cooperative Programme for Monitoring and Evaluation of the Long-Range Transmission of Air Pollutants in Europe (EMEP). A more

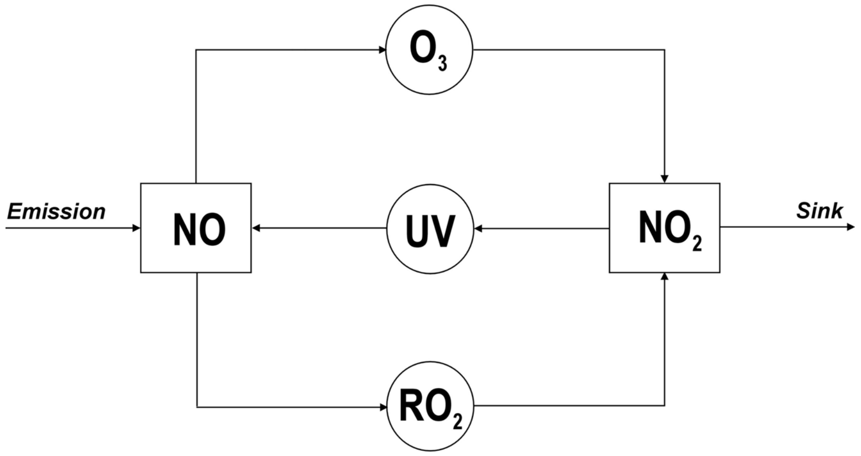

**FIGURE 1.** A scheme of interactions of NO, $NO_2$, $O_3$, and $RO_2$ leading to enrichment of the surface layer of the atmosphere with $O_3$.

complex ozone model was employed by the Meteorological Synthesizing Centre-West (MSC-W) of EMEP[7].

Penetration of ozone into plant cells via stomata negatively affects plant growth and productivity. A recent summary of the effects on a cell and organism level, as well as the estimates of ozone-caused reductions in plant productivity and respective losses, can be found in Semenov et al.[8,9,10]. Reductions in annual increment of tree biomass by 10% and more caused by anthropogenic ozone took place in Austria, Great Britain, Hungary, Germany, Greece, Netherlands, Poland, Slovak Republic, France, Czech Republic, Slovenia, and Switzerland in the 1990s. The annual losses of agricultural production due to anthropogenic ozone exceeded \$2 billion in Europe during this time[10].

Terrestrial plant response to enhancement of ozone content can be described by the following empirical model:

$$P = P_0 \exp (b \cdot \Delta C) \qquad (1)$$

where $P_0$ and $P$ are initial and altered values of plant productivity over the growing season, $\Delta C$ is the change in surface ozone concentration averaged over growing season, and $b$ is a model parameter[11]. An alternative approach, based on a cumulative index, so-called AOT40 (Accumulated Over Threshold 40 ppm), was developed and successfully applied by Fuhrer[12]. Model parameter $b$ for various groups of plants – in particular, for cereal crops and trees – was estimated through statistical analysis of published experimental dose-response data[9,10,11].

A fate of nitrogen oxides in the lower atmosphere is not only oxidation through chemical reactions with ozone, peroxyradicals, etc.; $NO_x$ and its derivatives (nitrites, nitrates, and nitric acid) are also removed from the atmosphere to the Earth's surface through wet and dry deposition. Assessments of atmospheric deposition were accomplished for oxidized nitrogen by MSC-W and MSC-E of EMEP[13] as well as by international research groups[4]. This comprises part of the routine activity of the EMEP MSCs.

Atmospheric $NO_x$ has no direct effect (i.e., through penetration into leaves) on higher plants[14]. However, enrichment of the lower atmosphere with $NO_x$ may positively affect plant growth and productivity as a result of subsequent increase in a flux of oxidized atmospheric nitrogen to the land surface. This pays a role of additional nitrogen fertilization of terrestrial plants. It is known that terrestrial plants are able to utilize soil nitrogen both as nitrate and ammonium[15,16], but particular species have individual preferences with respect to this. Since the subject of this paper is the large-scale average effect on plants, these differences will not be taken into account. Thus, the effect will be assumed to be dependent, on average, on the total amount of soil nitrogen accessible for plants.

The response of terrestrial plants to an increase in atmospheric deposition of nitrogen can be estimated on the basis of a cause-effect relationship. This relationship describes a relative change in plant productivity (crop yield or tree biomass increment) in response to a given change in the rate of nitrogen fertilizer applied. The following model was adopted to quantify such a relationship:

$$P = P_0 \exp (b \cdot \Delta F) \qquad (2)$$

where $P_0$ and $P$ are initial and altered values of plant productivity over growth season, $\Delta F$ is the additional amount of fertilizer per square unit applied during the season, and $b$ is a model parameter. Note the similarity of models (1) and (2).

Model parameter $b$ is estimated from the results of dose-response experiments published in scientific literature. This information is widely presented in periodicals, because the efficacy of nitrogen fertilization was and still is one of the major focuses of agricultural science. Results of such experiments quantitatively describe increases in crop yield and tree biomass increment in response to a given increase in application rate of nitrogen fertilizers. In this paper we used such information on wheat, *Triticum aestivum* L.[17], barley, *Hordeum vulgare* L.[18], corn, *Zea mays* L.[19], crested wheatgrass, *Agropyron cristatum* L.[20], and Douglas fir, *Pseudotsuga menziesii* (Mirb.) Franco[21].

In the experiments, plant response to a given addition in applied fertilizer was gradually declined along with an increase in initial application rate; that is, relative efficacy of each next unit of fertilizer applied was less than for the previous unit. This is a well-known saturation phenomenon. However, if one considers experimental data on the effects of applications with a rate less than 50 kg N/ha such saturation appears insignificant. Therefore, we adopted model (2) for this particular range.

For each application rate D*F* used in an experiment with a given plant species, the following formula (3) was employed in estimating model parameter *b*:

$$b = \left(\ln\left(P/P_0\right)\right)\big/\Delta F \qquad (3)$$

A value of this parameter for a plant species was further calculated as an average over all experiments with the given species.

Practically, terrestrial plants have access to oxidized nitrogen depositing to the land surface only during the growing season. Nitrates are very mobile in soil and easily migrate to ground waters or freshwater basins (streams, rivers, lakes). Therefore, atmospheric nitrogen accumulated by land surface over late fall to winter to early spring has little chance to be utilized by terrestrial plants. As a result of this circumstance in Europe and North America (where most problems with anthropogenic $NO_x$ have emerged in the mid and high latitudes), less than one third of deposited oxidized nitrogen is accessible to terrestrial plants.

One can estimate the anthropogenic component of oxidized nitrogen deposition through deduction of background (natural) values from the total flux of oxidized nitrogen from the atmosphere to the land surface. The percentage annual increase in plant productivity caused by the actual anthropogenic component of deposition of oxidized nitrogen can be calculated through dividing this component by three and further applying formula (2).

## RESULTS AND DISCUSSION

The long-term mean value of concentration of surface ozone measured at background monitoring stations from 1973 to 1992 were about 50 μg/$m^3$, and the annual means varied from 25.5–84.7 μg/$m^3$[10]. In the 1990s, in some regions of western and central Europe, ozone surface concentrations**,** averaged over the vegetative season, reached and sustained a level of 90 mg/$m^3$. Thus, the anthropogenic component of ozone content has become significant.

Surface ozone concentrations in Europe for the 1990s were calculated by MSC-E and presented in an annual report[13]. Excesses for these concentrations and their background levels averaged over the vegetative season have been calculated[8,11]. Coefficient *b* for model (1) was estimated[10,11] for several groups of crop and tree species. Conversion of the excesses into declines in plant productivity using formula (1) yielded the estimates of percentage damage to plants in Europe over the EMEP grid[10]. The damage varied from 10–15% in the Netherlands (maximal values), to 4–10% in Russia (minimal values). Estimation of coefficient *b* for model (2) using experimental data extracted from the scientific literature (see methods section) gave the values presented in Table 3.

The values in Table 3 actually reflect the percentage increase in plant seasonal productivity in response to an additional 1 kg N/ha in nitrogen fertilizer applied within a range of application rates of 0–50 kg N/ha.

It follows from Table 3 that an additional 1 kg N/ha applied within this range leads to about a 1% increase in crop productivity. This value is substantially more than the 0.21% calculated for the tree *Pseudotsuga menziesii* (Mirb.) Franco. Experimental data from a study by Miller and Tarrant[21] on 15-year experiments with a Douglas fir stand were employed for this estimation. However, it should be mentioned that the minimal fertilization rate applied in these treatments was 157 kg N/ha. Thus, an initial stage of saturation could have influenced the results.

**TABLE 3**
**Estimates of Model Parameter *b* of Model (2) for Some Plant Species (kg N/ha)$^{-1}$**

| Plant species | Estimate of *b* x 100 |
|---|---|
| *Agropyron cristatum* | 0.69 |
| *Hordeum vulgare* | 0.70 |
| *Triticum aestivum* | 1.30 |
| *Zea mays* | 1.13 |
| Mean of above values | 0.96 |
| *Pseudotsuga menziesii* | 0.21 |

According to an assessment by Ryaboshapko[4], annual nitrogen deposition over the territory of Russia varied from 40 to 400 mg N/$m^2$, that is, 0.4–4 kg N/ha. The lower limit of this range is typical of those regions of Russia where anthropogenic component of atmospheric nitrogen is definitely negligible because of the absence of big cities, industrial centers, or agricultural lands. Therefore 0.4 kg N/ha can be adopted as a continental background value for Euro-Asia. A maximum is reached in the Netherlands — 1500 mg N/$m^2$, that is, 15 kg N/ha[13].

Thus, for those regions in western Europe where nitrogen depositions are the highest — for example, 15 kg N/ha per annum in some regions of the Netherlands — respective increase in productivity of agricultural plants reaches [(15 – 0.4)/3] – 1% » 5%. In Russia, where maximum values of annual nitrogen deposition are less than 40 kg N/ha, respective increase in productivity does not exceed [(4 – 0.4)/3] – 1% = 1.2%.

These estimates are not valid for managed lands where nitrogen fertilizers are normally applied at typically high rates. The usual application rate of nitrogen fertilizers is 100–200 kg N/ha per season. In this connection the role of additional "atmospheric fertilization", even at a rate of 15 kg N/ha (as in the Netherlands), appears to be negligible because of the saturation effect.

Nazarov and Fridman[22] have given a fundamental analysis of the phenomenon of acidification of precipitation and soils due to $NO_x$ enrichment of the lower atmosphere. They provide convincing evidence that this process has not taken place over the former Soviet Union in the 20th century. The main reason for this was neutralization of acid compounds by alkaline particles emitted from anthropogenic sources along with the acid compounds. Thus, paradoxically, low technological level of purification systems in Russia at the time helped to avoid acidification of the environment. Therefore, no effects of a continental scale on plants can be expected.

However, even in western Europe, where quality of purification systems was much higher and therefore precipitation was more acid, no effects of a continental scale on plant productivity have been documented (although locally a certain damage definitely occurred). On the large-scale in Europe, acid deposition has seriously influenced some water ecosystems, predominantly in Scandinavia because of specific geochemical features of its lithosphere.

## CONCLUSIONS

Thus, anthropogenic emissions of $NO_x$ to the lower atmosphere predominantly affect productivity of terrestrial plants through enhancement of surface ozone content, and this effect is negative. The accompanying positive effect, through increase in deposition of oxidized nitrogen to soils, only mitigates the ozone effect to a modest degree. Effect of acidification of precipitation and soils on terrestrial plants appears to be insignificant on the large scale.

## ACKNOWLEDGEMENTS

The author would like to thank Vladimir Egorov (Institute of Global Climate and Ecology, Moscow), Elena Popova (Institute of Geography, Moscow) and Lynne Thompson (University of Arkansas at Monticello) for fruitful discussions on this paper.

| | | |
|---|---|---|
| Received: | July | 10, 2001 |
| Revised: | August | 17, 2001 |
| Accepted: | August | 17, 2001 |
| Published: | September | 24, 2001 |
